# Oral Corticosteroid Therapy in Lumbar Radiculopathy: Evidence From Randomized Controlled Trials

**DOI:** 10.7759/cureus.109327

**Published:** 2026-05-21

**Authors:** Vaishnavi Sharma, Hussein Akil, Neel Badhe, Rami Abou Faour, Zeinab Nahle, Monalisa Bhattacharyya, Chinedu Egu, Elie Najjar

**Affiliations:** 1 Department of Neurological Surgery, The Centre for Spinal Studies and Surgery, Nottingham University Hospitals NHS Trust, Nottingham, GBR; 2 Department of Neurological Surgery, Addenbrooke's Hospital, Cambridge University Hospitals NHS Foundation Trust, Cambridge, GBR; 3 Department of Orthopedic Surgery, American University of Beirut Medical Center, Beirut, LBN; 4 School of Medicine, University of Cambridge, Cambridge, GBR; 5 Department of Orthopedic Surgery and Traumatology, Faculty of Medicine, University of Balamand, Beirut, LBN; 6 Department of Orthopedics and Traumatology, Lebanese American University Medical Center, Beirut, LBN; 7 Department of Orthopedic Surgery, Addenbrooke’s Hospital, Cambridge University Hospitals NHS Foundation Trust, Cambridge, GBR; 8 Department of Spine Surgery, The Centre for Spinal Studies and Surgery, Queen's Medical Centre, Nottingham University Hospitals NHS Trust, Nottingham, GBR

**Keywords:** lumbar radiculopathy, lumbar spine disorders, oral steroids, randomized controlled trials, systematic review

## Abstract

Oral corticosteroids are frequently prescribed for lumbar radicular pain, despite uncertainty regarding their clinical benefit. Electronic databases, including MEDLINE, Embase, CENTRAL, Scopus, and PubMed, were searched from inception to identify randomized controlled trials assessing oral corticosteroids for lumbar radicular pain. The primary outcome was functional improvement, while secondary outcomes included pain intensity, quality of life, patient-reported improvement, need for surgery, and adverse events. Risk of bias was assessed using the Cochrane RoB 2 tool, and due to heterogeneity across studies, results were synthesized narratively. Four trials involving 369 participants were included. One placebo-controlled trial demonstrated a modest but statistically significant improvement in disability with oral corticosteroids, without a corresponding reduction in pain intensity. Two additional placebo-controlled trials found no significant between-group differences in pain or function. An active-comparator trial reported lower pain scores with oral corticosteroids compared with pregabalin or gabapentin, although interpretation was limited by methodological concerns. Quality-of-life outcomes were inconsistent, rates of surgery did not differ between groups, and oral corticosteroids were associated with more frequent short-term adverse events. Overall, oral corticosteroids may provide modest functional benefit in lumbar radiculopathy but do not consistently reduce pain or the need for surgery and are associated with increased short-term adverse effects, indicating that their use should be individualized with careful consideration of potential benefits and harms.

## Introduction and background

Lumbar radicular pain is a common and clinically significant presentation in spinal practice, frequently associated with lumbar disc herniation and nerve root compression. It is a major driver of functional limitation, healthcare utilization, and specialist referral, particularly during the acute and subacute phases of disease [1,2]. Initial management is typically nonoperative and multimodal, incorporating activity modification, structured exercise, and analgesic optimization. While surgical decompression is effective for selected patients with refractory or progressive symptoms, the majority are managed conservatively, creating a therapeutic window in which short-term pharmacological interventions are often considered [3-5].

Oral corticosteroids continue to be prescribed in this setting based on their anti-inflammatory effects and theoretical ability to reduce nerve root edema (swelling around the affected nerve root) and chemical radiculitis, an inflammatory response triggered by disc material contacting neural structures, associated with disc herniation [6]. Their ease of administration and noninvasive nature make them an attractive option compared with procedural interventions. However, systemic exposure carries recognized risks, and the clinical value of oral steroid therapy in altering pain, function, or downstream outcomes remains uncertain.

Randomized controlled trials evaluating oral corticosteroids for lumbar radicular pain have produced inconsistent results. Some studies report modest short-term improvements in pain or disability, while others demonstrate no meaningful benefit over placebo or active pharmacological comparators. Interpretation is further complicated by heterogeneity in study design, corticosteroid regimens, comparator choice, outcome measures, and follow-up duration [7-10]. As a result, there is no clear consensus regarding the role of oral corticosteroids in contemporary radicular pain management.

Despite ongoing use in routine clinical practice, the randomized controlled trial evidence specific to oral corticosteroids for lumbar radicular pain has not been synthesized in a focused and methodologically consistent manner. In particular, uncertainty persists regarding the magnitude and durability of any clinical benefit, the relevance of comparator choice, and the balance between symptomatic relief and adverse effects.

The aim of this systematic review is therefore to synthesize evidence from randomized controlled trials evaluating the efficacy and safety of oral corticosteroids, compared with placebo or active pharmacological comparators, in the treatment of lumbar radicular pain. The review addresses a clinically pragmatic question: do oral corticosteroids provide meaningful short- or long-term benefit in pain, function, or patient-reported outcomes sufficient to justify their use in routine spinal practice?

## Review

Methods

Study Design

This study was conducted as a systematic review in accordance with the PRISMA guidelines [11]. Quantitative synthesis was considered but ultimately not performed due to substantial heterogeneity across studies in terms of comparators, outcome measures, and reporting, precluding meaningful meta-analysis.

Data Sources and Search Strategy

A comprehensive literature search was conducted in MEDLINE, Embase, the Cochrane Central Register of Controlled Trials (CENTRAL), Scopus, and PubMed from inception to the date of the final search, January 2026.

The search strategy combined free-text terms and controlled vocabulary (MeSH and database-specific subject headings) related to lumbar radicular pain and oral corticosteroid therapy. Search terms included combinations of “sciatica”, “radiculopathy”, “radicular pain”, “lumbar radiculopathy”, “herniated disc”, “lumbar disc herniation”, “leg pain”, “oral steroid”, “oral corticosteroid”, “oral glucocorticoid”, “prednisone”, “prednisolone”, and “dexamethasone”. Search strategies were adapted for each database to maximize sensitivity.

Reference lists of all included studies and relevant reviews were manually screened to identify additional eligible trials. The search itself was not restricted by language; however, due to resource limitations, only studies published in English were included in the final review.

Eligibility Criteria

Eligibility criteria were defined using the PICOTS framework and are presented alongside exclusion criteria in Table 1.

**Table 1:** Inclusion and exclusion criteria ODI, Oswestry Disability Index; RMDQ, Roland-Morris Disability Questionnaire; SF-12, Short Form-12; SF-36, Short Form-36

Inclusion criteria	Exclusion criteria
Population: Adults with lumbar radicular pain (sciatica) attributed to lumbar disc herniation or nerve root compression, diagnosed clinically and/or radiologically	Non-randomized study designs, including observational, retrospective, or case series studies
Intervention: Systemic oral corticosteroid therapy, including prednisone, prednisolone, dexamethasone, or equivalent formulations	Studies involving patients with non-radicular low back pain
Comparator: Placebo or usual care without systemic corticosteroids (active comparators eligible for qualitative synthesis only)	Use of non-oral corticosteroid administration routes (e.g., epidural, intravenous, or intramuscular)
Outcome 1: Functional improvement measured using validated disability instruments (ODI or RMDQ)	Animal studies
Outcome 2: Pain intensity measured using a visual analogue scale or a numeric rating scale	Conference abstracts, editorials, letters, or narrative reviews
Outcome 3: Health-related quality of life measured using SF-36 or SF-12 physical and mental component summary scores	Publications in languages other than English
Outcome 4: Patient-reported global improvement or satisfaction	
Outcome 5: Need for surgical or procedural intervention	
Outcome 6: Adverse events	
Any follow-up duration	
Randomized controlled trials only	

Study Selection

All records identified through database searches were imported into a reference management software, and duplicates were removed. Two reviewers independently screened titles and abstracts for eligibility. Full-text articles were retrieved and assessed for all potentially relevant studies. Disagreements regarding inclusion were resolved through discussion and consensus. Reference lists of included studies were manually screened to identify additional eligible trials.

Data Extraction

Data extraction was performed independently by two reviewers using a standardized data collection form. Extracted data included study characteristics (first author, year of publication, country, study design, sample size, and follow-up duration); patient characteristics (mean age and sex distribution); intervention details (type of oral corticosteroid, dosing regimen, and treatment duration; comparator type and dose); and outcome data for all prespecified endpoints. Discrepancies were resolved by consensus.

Risk of Bias Assessment

Risk of bias was independently assessed by two reviewers using the Cochrane Risk of Bias 2 (RoB 2) tool for randomized controlled trials [12]. Five domains were evaluated: randomization process, deviations from intended interventions, missing outcome data, measurement of outcomes, and selection of reported results. Each domain was judged as low risk, some concerns, or high risk of bias, with an overall judgment assigned to each study. Disagreements were resolved by consensus.

Data Analysis

Given the heterogeneity of comparators, outcome instruments, and reporting across included studies, formal meta-analysis was not performed. Results were synthesized narratively, with outcomes reported using study-level effect estimates where available, including mean differences, risk ratios, confidence intervals, and p-values as reported in the original trials.

Results

Study Selection

**Figure 1:**
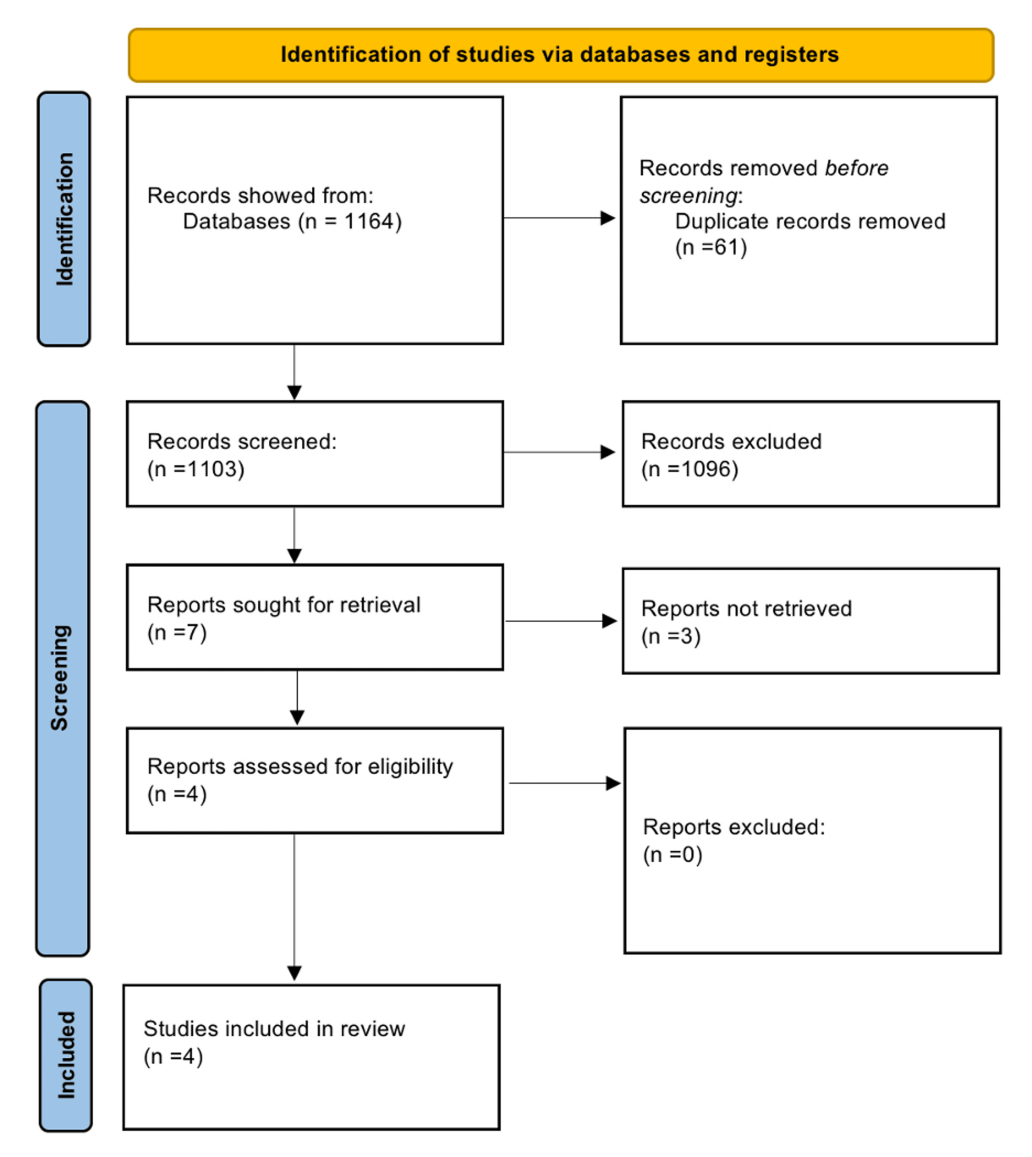
PRISMA flowchart

From 1103 unique records, seven full-text articles were reviewed, and four met the inclusion criteria. No additional eligible studies were identified from reference lists. The selection process is summarized in Figure 1.

Risk of Bias

**Figure 2:**
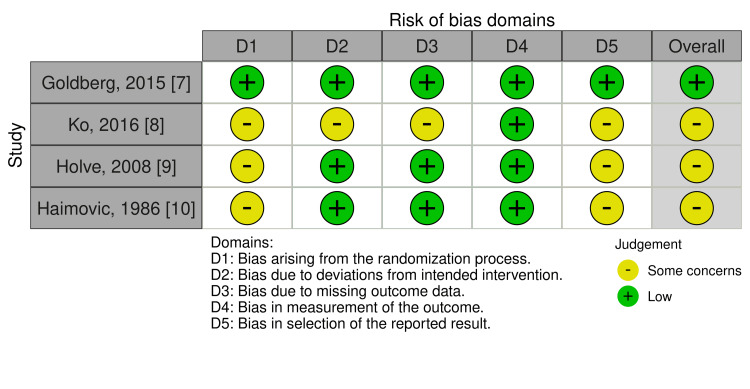
Risk of bias of included studies assessed using RoB 2 [7-10] RoB 2, Risk of Bias 2

Risk of bias was assessed using the Cochrane RoB 2 tool (Figure 2). One study [7] was judged to have a low risk of bias across all assessed domains. The remaining three studies [8-10] were judged as having some concerns overall, most commonly related to the randomization process and potential bias in the selection of the reported results. No study was judged to be at high risk of bias in any domain.

Study Characteristics

Four randomized controlled trials published between 1986 and 2016 were included [7-10], comprising 385 participants, of whom 369 were analyzed (Table 2). Overall, 235 participants received oral corticosteroids, and 134 received control treatment (placebo in three trials; pregabalin or gabapentin in one trial).

**Table 2:** Study characteristics NS, not significant; ODI, Oswestry Disability Index; PCS/MCS, physical/mental component summary; RCT, randomized controlled trial; RMDQ, Roland-Morris Disability Questionnaire; SF-12, Short Form-12; SF-36, Short Form-36

Study (year)	Country	Design	Setting	Sample size (analyzed)	Mean age (years)	Sex (M/F)	Steroid regimen	Comparator	Follow-up	Key outcomes reported
Goldberg et al. (2015) [7]	USA	Double-blind RCT	Outpatient	269	46.3	55%/45%	Prednisone 60 mg ×5 days → 40 mg ×5 days → 20 mg ×5 days (total 600 mg)	Placebo	3 weeks, 52 weeks	ODI, pain NRS, SF-36 PCS/MCS, global improvement, adverse events
Ko et al. (2016) [8]	South Korea	RCT (non-concealed allocation)	Outpatient	40	62.5	NS between groups	Triamcinolone 4 mg BID ×2 weeks (dose-adjusted)	Pregabalin or gabapentin	2, 6, 12 weeks	ODI, RMDQ, pain VAS, SF-36, satisfaction
Holve and Barkan (2008) [9]	USA	Double-blind RCT (quasi-randomized)	Outpatient	27	42.6	63%/37%	Prednisone 60 mg ×3 days → 40 mg ×3 days → 20 mg ×3 days	Placebo	Weekly to 6 months	RMDQ, pain scale, SF-12, return to work
Haimovic and Beresford (1986) [10]	USA	Double-blind RCT	Inpatient (bed rest)	33	Not reported	Not reported	Dexamethasone taper 64 mg → 32 mg → 16 mg → 12 mg → 8 mg (7 days)	Placebo	7 days; 1-4 years	Categorical pain scale, work status

Sample sizes ranged from 27 to 269 participants. Corticosteroid regimens included prednisone, dexamethasone, or triamcinolone, with treatment durations of seven to 15 days. Mean age, reported in three trials, was 46.8 years in the steroid group and 49.2 years in controls (p = 0.11); no between-group age or sex differences were observed where reported. Follow-up ranged from three weeks to 52 weeks. Full study characteristics are shown in Table 2.

Functional Outcomes

Functional outcomes were assessed using validated disability instruments in three trials [7-9], although numerical between-group data were available from only two studies [7,8]. In the largest placebo-controlled trial [7], oral corticosteroids were associated with significantly greater functional improvement than placebo at both assessed time points. At three weeks, the mean difference in Oswestry Disability Index (ODI) scores [13] was -5.64 points (95% CI -6.75 to -4.53; p < 0.0001), and this difference persisted at 52 weeks (mean difference -7.46 points, 95% CI -8.79 to -6.13; p < 0.0001). Baseline ODI scores were comparable between groups. In contrast, in the trial comparing oral corticosteroids with pregabalin [8], no statistically significant between-group differences in ODI were observed at six weeks (mean difference -2.4, p = 0.27) or 12 weeks (mean difference 0.9, p = 0.71).

In a smaller outpatient placebo-controlled trial [9], disability was assessed using the Roland-Morris Disability Questionnaire (RMDQ) [14]; however, endpoint numerical values were not reported, and no statistically significant between-group differences were demonstrated. The inpatient placebo-controlled trial [10] did not employ a standardized disability instrument and reported no between-group differences in functional status as assessed by work participation.

Pain Outcomes

In the placebo-controlled trial by Goldberg et al. [7], there was no statistically significant difference between prednisone and placebo in change in below-waist pain measured using a numeric rating scale at three weeks (adjusted difference -0.3, 95% CI -1.0 to 0.4; p = 0.34) or 52 weeks (adjusted difference -0.6, 95% CI -1.3 to 0.2; p = 0.15).

In Holve and Barkan [9], pain was assessed using the Roland-Morris Pain Rating Scale. The authors reported no statistically significant between-group differences in pain at any assessed time point. Within-group improvement from baseline was observed in the prednisone arm beginning at week 1, whereas within-group improvement in the placebo arm did not reach statistical significance until week 4 (except week 2). Numerical endpoint values were not reported.

In Haimovic and Beresford [10], pain was assessed using a 7-point categorical pain scale (0 = no pain; 6 = severe constant pain). Early improvement at seven days, defined as pain being “definitely less” than baseline, occurred in 7/21 patients receiving dexamethasone and 4/12 receiving placebo for resting pain, and in 8/19 and 1/6 patients, respectively, for pain provoked by straight-leg raising. None of these between-group differences was statistically significant on chi-square testing. At long-term follow-up (one to four years), sustained improvement (pain score ≤3) was observed in 8/16 dexamethasone-treated patients and 7/11 placebo-treated patients, with no between-group difference.

In the active-comparator trial by Ko et al. [8], leg pain measured using a 10-point visual analogue scale was lower in the oral corticosteroid group than in the pregabalin or gabapentin group at 2 weeks (3.8 ± 2.8 vs 4.3 ± 2.4; p < 0.001), 6 weeks (2.0 ± 2.3 vs 3.0 ± 2.2; p = 0.001), and 12 weeks (2.0 ± 2.6 vs 3.2 ± 2.2; p < 0.001).

Health-Related Quality of Life

Health-related quality of life was assessed using generic validated instruments in three trials [7-9]. In the placebo-controlled trial by Goldberg et al. [7], quality of life was measured using the Short Form-36 (SF-36) [15]. At three weeks, the Physical Component Summary score was higher in the prednisone group than in the placebo group (between-group difference 3.3 points, p = 0.001), while no difference was observed in the Mental Component Summary score. At 52 weeks, no between-group difference was observed in the Physical Component Summary score; however, the Mental Component Summary score was higher in the prednisone group (between-group difference 3.6 points, p = 0.02).

In the outpatient placebo-controlled trial by Holve and Barkan [9], quality of life was assessed using the Short Form-12 (SF-12) [16]. No statistically significant between-group differences were reported in either physical or mental health scores at any assessed time point. Numerical endpoint values were not reported.

In the active-comparator trial by Ko et al. [8], quality of life was assessed using the SF-36. Greater improvement in physical component scores was reported in the corticosteroid group compared with pregabalin or gabapentin at follow-up, while no consistent between-group differences were observed in mental health component scores. Group-level numerical values were not consistently reported.

Patient-Reported Global Improvement

Goldberg et al. [7] assessed global change using a seven-category ordered patient global assessment of leg-pain change. At three weeks, 147/179 (82.1%) of participants in the prednisone group reported being at least “somewhat better” compared with 61/88 (69.3%) in the placebo group (RR 1.2, 95% CI 1.0-1.4; p = 0.02). At 52 weeks, the proportion reporting at least “somewhat better” was 143/157 (91.1%) with prednisone versus 66/77 (85.7%) with placebo (RR 1.1, 95% CI 1.0-1.2; p = 0.30).

Ko et al. [8] reported patient-rated objective improvement at 2, 6, and 12 weeks. Mean improvement scores were 2.3 ± 0.9, 2.4 ± 1.0, and 2.3 ± 1.2 in the steroid group versus 2.0 ± 0.7, 2.1 ± 0.9, and 2.0 ± 0.8 in the pregabalin or gabapentin group, with no statistically significant between-group difference (p = 0.657). Physician-rated objective improvement similarly did not differ between groups (p = 0.748).

Satisfaction

Assessed only by Ko et al. [8], patient satisfaction did not differ between oral corticosteroids and pregabalin at 12 weeks (mean difference -0.3, p = 0.37).

Need for Surgery

Goldberg et al. [7] prespecified lumbar spine surgery incidence as a secondary outcome. At three weeks, surgery had occurred in 51/179 (28.4%) patients in the prednisone group and 23/88 (26.1%) in the placebo group (adjusted RR 1.1, 95% CI 0.8-1.7; p = 0.52). At 52 weeks, surgery had occurred in 18/179 (9.9%) versus 8/88 (9.1%), respectively (adjusted RR 1.2, 95% CI 0.5-2.6; p = 0.68).

Holve and Barkan [9] reported that one patient in the control group underwent L5 discectomy, with no surgical interventions reported in the prednisone group.

Haimovic and Beresford [10] reported that six dexamethasone-treated patients who did not experience sustained improvement subsequently underwent lumbar laminectomy; the number of surgical interventions in the placebo group was not explicitly reported, precluding between-group comparison.

Procedural Intervention

Holve and Barkan [9] reported that epidural steroid injections were required in 2/13 (15.4%) patients in the prednisone group and 6/14 (42.9%) patients in the placebo group; this difference did not reach statistical significance (p = 0.12).

Adverse Events

Goldberg et al. [7] collected adverse events at each study contact and reported detailed events through the three-week visit. By three weeks, at least one adverse event was reported by 88/179 (49.2%) participants in the prednisone group versus 21/88 (23.9%) in the placebo group (p < 0.001). Events occurring more frequently with prednisone included insomnia (26.0% vs 10.2%, p = 0.003), nervousness (18.2% vs 8.0%, p = 0.026), and increased appetite (22.1% vs 10.2%, p = 0.018). Other adverse events, including headache, indigestion, joint pain, and sweating, did not differ significantly between groups.

By 52 weeks, 208/269 (77.3%) participants reported a total of 723 adverse events. There were no between-group differences in the mean number of adverse events per person (2.70 vs 2.69; p = 0.98) or in the proportion reporting at least one adverse event (80.1% vs 71.6%; p = 0.12). Five serious adverse events occurred over 52 weeks (three in the prednisone group and two in the placebo group), and none was judged likely related to study medication.

Holve and Barkan [9] reported no clinically significant adverse events related to medication in either group.

Discussion

Functional Outcomes

This systematic review synthesized evidence from four randomized controlled trials evaluating oral corticosteroids for lumbar radicular pain [7-10]. Across placebo-controlled evidence, oral corticosteroids were associated with modest improvements in disability and short-term patient-perceived recovery, while pain intensity did not differ from placebo, and surgery rates were unchanged [7]. Evidence from an active-comparator trial suggested lower leg pain scores with oral corticosteroids compared with pregabalin or gabapentin, but this comparison addresses a different clinical question and should be interpreted separately from placebo-controlled trials [8].

Functional improvement represented the most consistent signal supported by placebo-controlled evidence. In the largest trial, prednisone improved ODI scores relative to placebo at both three weeks and 52 weeks [7]. However, the magnitude of between-group separation warrants cautious interpretation. Evidence syntheses in radiculopathy commonly consider a 10-point ODI change and a 5-point RMDQ change as minimum clinically important difference thresholds, placing the observed ODI differences in a modest range rather than clearly clinically transformative [17]. The smaller outpatient placebo-controlled trial using the RMDQ did not demonstrate a between-group functional advantage and did not report extractable numerical values, limiting corroboration of the functional signal [9]. Overall, current evidence supports the possibility of a small functional benefit, but this conclusion remains largely driven by a single well-conducted study [7].

Pain Outcomes

Pain outcomes were less supportive. In the largest placebo-controlled trial, prednisone did not reduce pain intensity compared with placebo at either short- or long-term follow-up [7]. This finding was consistent with older placebo-controlled trials that also failed to demonstrate statistically significant between-group pain differences using alternative pain scales [9,10]. The dissociation between disability and pain outcomes suggests that any benefit of oral corticosteroids may relate more to activity tolerance or symptom appraisal than to direct analgesia, reinforcing that pain relief should not be the primary expectation of treatment [7].

Active-comparator evidence should be interpreted with particular caution. Ko reported lower leg pain VAS scores with oral corticosteroids compared with pregabalin or gabapentin at multiple time points [8]. However, non-concealed allocation, heterogeneous comparators, and post-randomization dose adjustment limit causal inference. These findings suggest a possible comparative advantage over specific neuropathic agents in that trial context but do not establish a consistent analgesic effect versus placebo [7,8].

Health-Related Quality of Life

Health-related quality-of-life outcomes were variably reported and did not materially strengthen evidence for sustained benefit. Goldberg et al. [7] observed short-term improvement in SF-36 physical component scores and a later difference in mental component scores, while other domains and time points showed no consistent separation. Holve et al. [9] reported no SF-12 differences, without numerical endpoints. Ko et al. [8] described greater improvement in SF-36 physical component scores with oral corticosteroids, but mental health outcomes were inconsistent and incompletely reported. Taken together, health-related quality-of-life findings suggest limited, domain-specific effects rather than broad or durable benefits [7-9].

Patient-Reported Recovery

Patient-reported global improvement provides context for these findings. In Goldberg et al. [7], a greater proportion of prednisone-treated participants reported being at least “somewhat better” at three weeks, but this difference was not sustained at 52 weeks. This pattern supports short-term perceived acceleration of recovery without durable separation in pain or downstream outcomes. In Ko et al. [8], patient and physician-rated objective improvement did not differ between groups despite lower pain scores with oral corticosteroids, suggesting a limited impact on the global indication of recovery.

Surgery and Escalation of Care

Downstream outcomes related to escalation of care were not improved. Surgery rates did not differ between prednisone and placebo in the largest trial [7]. Other trials reported sparse and inconsistently defined surgical endpoints that precluded reliable comparison [9,10]. Collectively, available evidence does not support oral corticosteroids as a strategy to reduce the likelihood of surgery [7,9,10].

Safety

Safety findings indicate a predictable short-term trade-off. Prednisone was associated with a higher frequency of early adverse events, including insomnia, nervousness, and increased appetite, while longer-term adverse event rates and serious adverse events were similar between groups [7]. Harms reporting in smaller trials was limited [9]. Given the modest magnitude of benefit, short-term adverse effects are clinically relevant when considering treatment [7].

These findings align with broader evidence syntheses of corticosteroid therapy for radicular pain. A Cochrane review of systemic corticosteroids concluded that effects are generally small, outcome-dependent, and of limited durability [17]. Similarly, a large evidence synthesis of epidural corticosteroid injections reported small, short-term improvements below clinically important thresholds and no sustained reduction in surgery risk [18]. Although oral and epidural routes are not directly comparable, the convergence of conclusions across delivery methods supports a cautious interpretation of corticosteroid efficacy in radiculopathy [17,18].

Strengths and Limitations

This review has several strengths, including its focused evaluation of oral corticosteroid therapy specifically for lumbar radicular pain and inclusion of randomized controlled trials only. However, the review is limited by the available primary literature. Only four trials met eligibility criteria, with heterogeneity in interventions, comparators, outcome instruments, and reporting quality precluding meta-analysis [7-10]. Incomplete numerical reporting constrained quantitative synthesis, and risk-of-bias assessment identified some concerns in three of four trials [8-10]. Variation in outcome measures and follow-up duration also limited consistency across studies and complicated the interpretation of the overall magnitude of treatment effect [7-10].

Clinical Implications and Future Directions

In practical terms, oral corticosteroids may provide modest improvement in disability and short-term perceived recovery in lumbar radiculopathy but do not consistently reduce pain intensity versus placebo, do not improve long-term quality of life, and do not reduce surgery rates while increasing short-term adverse effects [7-10]. Their use should therefore be individualized, with realistic expectations and explicit discussion of benefits and harms.

Future trials should be placebo-controlled, adequately powered, and reported to contemporary standards, with prespecified primary outcomes, complete numerical reporting, standardized escalation endpoints, and robust harm capture to clarify whether specific patient subgroups derive clinically meaningful benefit [18].

## Conclusions

The available randomized controlled trial evidence indicates that oral corticosteroids may provide modest short-term improvements in pain and function for patients with lumbar radicular pain, but these benefits are not sustained over the long term. Importantly, oral steroids do not appear to reduce the need for surgical intervention and are associated with a higher incidence of temporary adverse effects. Given these findings, oral corticosteroids should not be considered a first-line therapy for radicular pain but may have a limited role in carefully selected patients with severe, acute symptoms who require short-term symptomatic relief. Future research should focus on large, well-designed trials comparing oral steroids with active first-line treatments and aim to identify patient subgroups most likely to benefit, ultimately enabling a more personalized and evidence-based approach to management.
